# Sustainable Hydrogels for Medical Applications: Biotechnological Innovations Supporting One Health

**DOI:** 10.3390/gels11070559

**Published:** 2025-07-21

**Authors:** Silvia Romano, Sorur Yazdanpanah, Orsolina Petillo, Raffaele Conte, Fabrizia Sepe, Gianfranco Peluso, Anna Calarco

**Affiliations:** 1 Research Institute on Terrestrial Ecosystems (IRET)-CNR, Via Pietro Castellino 111, 80131 Naples, Italy; silvia.romano@iret.cnr.it (S.R.); soruryazdanpanah@cnr.it (S.Y.); orsolina.petillo@cnr.it (O.P.); gianfranco.peluso@cnr.it (G.P.); anna.calarco@cnr.it (A.C.); 2 Department of Experimental Medicine, University of Campania “Luigi Vanvitelli”, Via Santa Maria di Costantinopoli 16, 80138 Naples, Italy; 3 National Biodiversity Future Center (NBFC), 90133 Palermo, Italy; 4 Faculty of Medicine and Surgery, Saint Camillus International University of Health Sciences, Via di Sant’Alessandro 8, 00131 Rome, Italy

**Keywords:** One Health, sustainable hydrogels, biocompatible polymers, microbial fermentation, recombinant proteins

## Abstract

The One Health paradigm—recognizing the interconnected health of humans, animals, and the environment—promotes the development of sustainable technologies that enhance human health while minimizing ecological impact. In this context, bio-based hydrogels have emerged as a promising class of biomaterials for advanced medical applications. Produced through biotechnological methods such as genetic engineering and microbial fermentation, these hydrogels are composed of renewable and biocompatible materials, including recombinant collagen, elastin, silk fibroin, bacterial cellulose, xanthan gum, and hyaluronic acid. Their high water content, structural tunability, and biodegradability make them ideal candidates for various biomedical applications such as wound healing, tissue regeneration, and the design of extracellular matrix (ECM)-mimicking scaffolds. By offering controlled mechanical properties, biocompatibility, and the potential for minimally invasive administration, sustainable hydrogels represent a strategic innovation for regenerative medicine and therapeutic interventions. This review discusses the characteristics and medical applications of these hydrogels, highlighting their role in advancing sustainable healthcare solutions within the One Health framework.

## 1. Introduction

**Figure 1:**
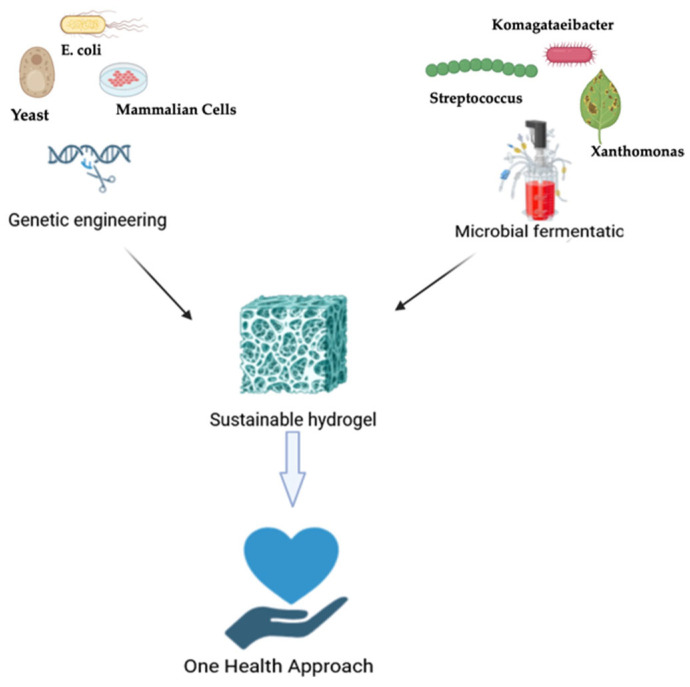
Schematic overview of biotechnological production paths for sustainable hydrogels under the One Health framework. Recombinant proteins are obtained via genetic engineering in microbial or mammalian cells, whereas microbial polysaccharides are produced by fermentation. Both approaches promote sustainable hydrogel design for biomedical applications such as wound healing, tissue regeneration, and drug delivery.

The concept of One Health has emerged as a critical paradigm in modern science and public health, emphasizing the intricate and inseparable relationships between human, animal, and environmental health. Rather than addressing health threats in isolated silos, the One Health approach promotes a transdisciplinary and collaborative model that integrates multiple sectors—including medicine, veterinary science, environmental science, epidemiology, and public policy—to address complex health challenges at the human–animal–ecosystem interface [1,2]. This comprehensive framework is essential in the face of mounting global pressures, including urbanization, habitat fragmentation, climate change, and increased interactions between humans, animals, and ecosystems. It promotes a systems-level understanding of how the health of living beings and natural systems are intimately intertwined. Environmental degradation, resource depletion, and unsustainable industrial practices have profound consequences on both animal and human health, often compromising the safety of water, food, and air [3]. In this light, One Health provides an essential framework for developing sustainable technologies and interventions that offer co-benefits across ecological, veterinary, and medical contexts. The One Health concept not only highlights the interconnection of human, animal, and environmental health, but it also directs the development of biomaterials that follow sustainability and biosafety guidelines. One Health advocates for the use of renewable biological systems such as genetically altered microorganisms and controlled microbial fermentation to manufacture sophisticated biomaterials such as hydrogels in place of animal-derived and petroleum-based chemicals [4,5]. At the core of this approach is the recognition that sustainable innovation—especially in the development of materials and therapeutics—must minimize harm to ecosystems while enhancing health outcomes across species [6]. However, implementing the One Health framework faces several practical challenges that limit its widespread adoption and effectiveness. Key barriers include the fragmentation of regulatory policies across human, animal, and environmental health sectors, which hinders coordinated action and policy enforcement. Additionally, limited interdisciplinary collaboration among scientists, clinicians, veterinarians, and environmental experts slows the integration of holistic solutions. Financial constraints, especially in low-resource settings, further restrict the deployment of comprehensive One Health strategies [7]. Engineered hydrogels offer promising solutions to mitigate these barriers by serving as versatile platforms across domains. In healthcare, they enable low-toxicity and sustained drug delivery applicable to both human and veterinary medicine, improving treatment efficacy and reducing side effects [8]. In agriculture, hydrogels can be used for the controlled release of biopesticides and fertilizers, enhancing crop protection while minimizing ecological impact. In environmental applications, their capacity to adsorb heavy metals, pharmaceuticals, and other pollutants positions them as effective tools for water and soil remediation [9]. This review aims to provide a comprehensive overview of sustainable hydrogel systems obtained through biotechnological routes, highlighting their molecular design, biomedical applications, and relevance to the One Health framework (Figure 1). A particular focus is placed on the dual role of these materials in enhancing therapeutic performance while minimizing environmental and biological risks. To guide the reader through the diverse typologies of bio-based hydrogels, the following sections are organized according to their biosynthetic origin—distinguishing hydrogels derived from genetic engineering of proteins (Section 3) from those obtained by microbial fermentation of polysaccharides (Section 4). Each of the materials addressed in this review exemplifies the One Health idea through sustainable sourcing and minimal environmental impact. For example, recombinant collagen and elastin eliminate the requirement for animal-derived tissues, reducing zoonotic risk and ethical problems [10]. Microbial-derived polysaccharides such as bacterial cellulose, xanthan gum, and hyaluronic acid can be made from agro-industrial waste, reducing environmental pollution and improving circular bioeconomy practices [11]. Moreover, the hydrogels explored in this work are characterized by high bio-sustainability. Unlike traditional synthetic hydrogels, which often rely on petrochemical sources, pose degradation challenges, and contribute to long-term environmental burden, hydrogels derived from engineered proteins or microbial fermentation offer a renewable and degradable alternative [12]. Then, early research on biotechnologically derived hydrogels has played a pivotal role in the development of hydrogel systems for drug delivery and tissue engineering. These studies demonstrated the potential of these materials to build biocompatible, biodegradable, and highly hydrated networks capable of encapsulating bioactive compounds and supporting cell growth [13]. For example, hydrogels engineered from recombinant proteins can be tailored to exhibit specific structural and functional properties while avoiding the ethical and environmental drawbacks associated with animal-derived or petroleum-based materials [14]. Similarly, hydrogels obtained through controlled fermentation of sustainable biomasses, such as polysaccharides or protein-rich microbial cultures, provide scalable and low-impact solutions with minimal ecological footprint [15]. The integration of these sustainable hydrogels into One Health strategies provides multiple advantages. In medicine, they enable precise, localized delivery of natural therapeutics—such as plant-based anti-inflammatory agents, antimicrobials, and wound-healing compounds—thereby reducing systemic side effects and improving patient outcomes [16]. In agriculture, they can be applied as carriers for biopesticides, biofertilizers, and plant stimulants, supporting crop health while reducing reliance on synthetic agrochemicals that often leach into ecosystems [17]. In environmental remediation, hydrogel systems can be designed to trap pollutants or release neutralizing agents in a controlled manner, contributing to ecosystem restoration [18]. Through these multi-sectoral applications, sustainable hydrogels serve as a unifying technological solution that simultaneously addresses the needs of people, animals, and the environment. Furthermore, the adoption of such biodegradable and biocompatible delivery platforms aligns with global policy directions spearheaded by organizations like the World Health Organization (WHO), the Food and Agriculture Organization (FAO), and the World Organization for Animal Health (WOAH). These agencies increasingly advocate for integrative, cross-sectoral solutions that build resilient health systems while preserving environmental integrity [19]. By embedding sustainability into the very materials used for therapeutic and agricultural interventions, the development of biosourced hydrogels helps operationalize the core principles of the One Health paradigm.

## 2. The Imperative for Hydrogel-Based Sustainable Therapeutic Strategies

Within the One Health framework, the development of sustainable and biocompatible hydrogel-based delivery systems represents a strategic convergence of therapeutic innovation, ecological responsibility, and global public health resilience. Hydrogels—three-dimensional, hydrophilic polymeric networks capable of retaining large volumes of water—offer a uniquely versatile platform for the encapsulation and controlled release of bioactive molecules [20]. Their high water content mimics natural biological tissues, enhancing biocompatibility and reducing the risk of adverse immune reactions [20]. This makes them particularly suitable for the administration of delicate natural compounds, such as polyphenols, flavonoids, terpenoids, and alkaloids, which often suffer from poor bioavailability, chemical instability, and rapid degradation when delivered in conventional formulations [21]. One of the key advantages of hydrogel systems is their ability to provide controlled and sustained drug release, thereby improving therapeutic efficacy and reducing the frequency of administration. This not only enhances patient compliance, especially in chronic disease settings, but also minimizes fluctuations in drug concentration that can lead to side effects or suboptimal treatment outcomes [21]. From a One Health standpoint, the ability to use lower drug doses over longer periods reduces the environmental load of pharmaceuticals, particularly those excreted unmetabolized or disposed of improperly, which can contaminate soil, water bodies, and animal habitats [22]. Hydrogels can also be engineered to respond to specific physiological stimuli such as pH, temperature, redox state, and enzymatic activity. These so-called “smart” or stimuli-responsive hydrogels allow for site-specific, on-demand drug release, which is highly valuable in treating localized inflammation, infections, or tumors [23]. This targeted delivery approach further reduces systemic exposure and collateral damage to healthy tissues, aligning with the One Health principle of minimizing unintended cross-species and ecological consequences of medical interventions. Beyond drug delivery, sustainable hydrogels are being increasingly explored as bioactive matrices for tissue engineering applications, acting as extracellular matrix (ECM) analogs to support cell adhesion, migration, proliferation, and differentiation. Their structural and biochemical tunability allows them to guide tissue regeneration processes in situ. Sustainable hydrogels can be engineered to exhibit ECM-like architecture, thereby enhancing tissue-specific interactions [24]. These systems are particularly promising for cartilage, bone, cardiac, and nerve regeneration. From a sustainability perspective, the source and synthesis of hydrogels are critical in determining their environmental impact. Recent advances in biotechnology now enable the fabrication of hydrogels using genetically engineered proteins (such as recombinant collagen, elastin, silk fibroin), which mimic the extracellular matrix and provide tailored mechanical and biochemical properties [25]. These protein-based hydrogels are biodegradable, non-toxic, and can be precisely modified to suit specific biomedical applications [25]. Another biotechnological approach involves the controlled microbial fermentation of renewable biomaterials to produce exopolysaccharides such as bacterial cellulose, xanthan gum, and hyaluronic acid. These microbial-derived polymers are inherently biodegradable, biocompatible, and non-immunogenic, making them ideal for eco-conscious hydrogel design [26]. Fermentation processes are also scalable, reproducible, and less resource-intensive compared to traditional chemical synthesis, thereby reducing the carbon and energy footprint associated with hydrogel production [26]. Both types of hydrogels possess high tunability in physical and mechanical properties—such as porosity, stiffness, degradation rate, and swelling behavior—allowing for precise control over drug-loading, release kinetics, and interaction with target tissues [27]. Finally, by enhancing the bioavailability and efficacy of bioactive compounds, hydrogel systems can contribute to the valorization of underutilized natural resources, including agricultural byproducts and traditional medicinal plants—thereby promoting biodiversity, local economic development, and cultural sustainability. Hydrogel-based technologies, however, have several key market limitations that continue to hinder their broader adoption. Scalability remains a critical challenge, particularly in the manufacturing of complex hydrogels from recombinant proteins, which often involves labor-intensive and cost-prohibitive biotechnological processes [28]. Regulatory approval represents another major barrier, as hydrogel formulations intended for clinical, veterinary, or environmental use must meet rigorous and often divergent safety, efficacy, and environmental impact standards. Additionally, the performance of hydrogels can vary significantly under different environmental conditions (e.g., temperature, humidity, pH), complicating their consistent application and commercialization. These constraints underscore the need for robust, reproducible, and adaptable biomaterials. Biotechnologically based hydrogels address many of these issues by offering tunable mechanical properties, enhanced biocompatibility, and precise control over degradation and release kinetics [29,30].

## 3. Protein-Based Hydrogels Obtained Through Genetic Engineering

**Figure 2:**
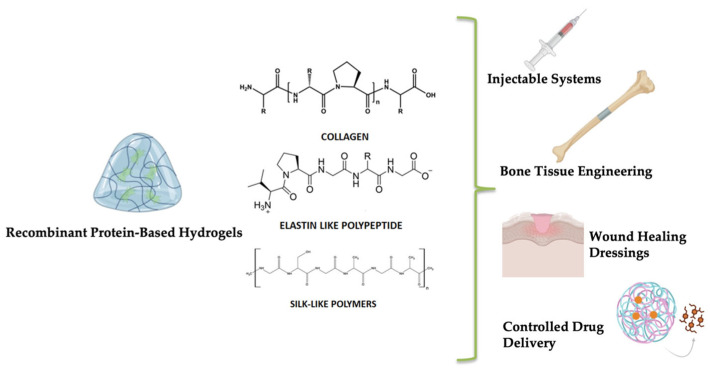
Overview of biomedical applications of hydrogels derived from recombinant proteins.

Inspired by natural systems, scientists are increasingly harnessing the precision of cellular protein synthesis machinery to engineer sustainable protein-based polymers (PBPs) with high structural and functional complexity [31]. Built from natural or engineered amino acid sequences, these genetically tailored proteins offer unique advantages over synthetic polymers—most notably, their uniform composition, excellent biocompatibility, biodegradability without toxic byproducts, and enzyme-responsive surface degradation [32]. Importantly, PBPs can be processed under mild, aqueous conditions to form hydrogel systems suitable for biomedical applications, such as targeted drug and gene delivery, tissue engineering, and regenerative therapies [33]. Genetic engineering enables tight control over polymer length, sequence, and functionality, allowing the construction of protein libraries with tunable mechanical, chemical, and biological properties. Through recombinant technologies, diverse functional motifs can be integrated into polymer backbones, enabling precise interactions with cells, biomolecules, or the extracellular matrix. Hydrogels developed through genetic engineering of sustainable proteins rely on the recombinant expression of structural proteins, including collagen, elastin, and silk-like polymers (Figure 2). These materials are created using controlled expression systems (e.g., *E. coli*, *P. pastoris*), which allow for fine customization of sequence, mechanical characteristics, and bioactivity [34]. Protein-based hydrogels are highly biocompatible and customizable, making them excellent for enhanced regenerative medicine and tailored drug delivery [35,36]. Their highly repetitive amino acid sequences promote self-assembly into well-defined secondary and tertiary structures, imparting mechanical strength, elasticity, and biological signaling capacity [37]. These features make them ideal candidates for constructing dynamic, stimuli-responsive hydrogels that support cellular function and tissue regeneration [37]. Importantly, the development of hydrogels through sustainable protein engineering aligns closely with the principles of the One Health framework. By relying on microbial biosynthesis and biodegradable protein materials, this approach reduces environmental burden, minimizes reliance on petrochemical-derived polymers, and supports the creation of safer, more sustainable biomedical devices.

### 3.1. Collagen

Collagen, the most abundant protein in the extracellular matrix (ECM) of animal tissues, is crucial for maintaining structural integrity and regulating cellular behavior [38]. Among the 29 known types, collagen types I, II, and III constitute over 90% of the total collagen in the human body. It provides tensile strength and supports the structure of skin, bones, cartilage, and connective tissues [39]. Each collagen α-chain is composed of repeating Gly-X-Y sequences, where the X and Y positions can be occupied by various amino acids. However, the presence of glycine at every third position is essential, as it enables the α-chains to fold into a tightly packed triple-helix structure [40]. Due to its biocompatibility, biodegradability, low immunogenicity, and affordability, collagen is widely used in biomaterials for drug delivery, tissue engineering, wound healing, and cosmetics [41]. However, collagen derived from animal sources poses challenges such as inconsistent quality, allergic reactions, and the risk of transmitting pathogens. Moreover, ethical concerns are associated with animal-sourced collagen [42]. Recombinant collagen offers a safer and more reliable alternative [43]. Produced through advanced in vitro techniques, recombinant collagen closely mimics natural human collagen by incorporating key post-translational modifications like hydroxylation and glycosylation [44]. Several types, including I, II, III, and V, have been successfully produced using gene expression systems. Depending on their α-chain composition, recombinant collagens can form homotrimers (e.g., types I–III), heterotrimers (e.g., type XI), or hybrid forms (e.g., type IX) [45]. Type III collagen, although less abundant than type I, plays a key role in maintaining tissue architecture and regulating scar formation. It constitutes approximately 8–11% of the dermal ECM, and its levels decline with age [10]. Because of its regenerative potential, type III collagen is increasingly used in skin repair therapies [46]. Microbial systems like *E. coli* and yeast are commonly used for large-scale recombinant collagen production. While bacterial hosts are cost-effective, they lack the enzymatic machinery for post-translational modifications. Yeast systems, though more complex, yield collagen that more closely resembles native human collagen [47]. This form of collagen offers a consistent supply of purified material free from animal components. One of the key benefits of using hydrogels obtained from genetic engineering of collagen is their excellent biocompatibility, along with optimal chemical and mechanical characteristics that make them suitable for mimicking the extracellular matrix of injured tissues [48]. In particular, collagen offers structural support because it forms a triple-helical fiber network that provides tensile strength and stability to tissues. Its highly organized structure mimics the natural extracellular matrix (ECM), enabling it to maintain the mechanical integrity of hydrogels and scaffolds. Additionally, collagen interacts with cell surface receptors to support adhesion, migration, and proliferation, further enhancing tissue regeneration [48]. These properties facilitate cell infiltration and nutrient exchange, both essential for promoting osteoinduction and osteogenic differentiation [48]. Rodrìguez-Cabello et al. modified a recombinant type I collagen protein (RCPhC1) with different chemical groups—methacrylamide (RCPhC1-MA), norbornene (RCPhC1-NB), and thiol (RCPhC1-SH)—to make it suitable for high-resolution 3D printing using a technique called two-photon polymerization (2PP). This advanced 3D printing method can create very detailed structures at a sub-micrometer scale. Although the results were only tested in the lab (in vitro), where cells were able to grow and multiply on these scaffolds, the study marked a significant step forward in using recombinant collagen for 3D-printed tissue engineering applications [49]. Xu et al. demonstrated that exosomes derived from mesenchymal stem cells (MSC-EVs) were incorporated into nanoparticles synthesized using recombinant type III collagen. These MSC-EVs facilitated the polarization of macrophages toward the M2 phenotype by modulating the miR-223/pKNOX1 signaling axis, thereby reducing inflammation and supporting skin wound repair. Both in vitro and in vivo experiments confirmed that the hydrogel composed of rhCol III and MSC-EVs has biomimetic properties, effectively regulated inflammation, promoted cell migration, and enhanced angiogenesis within the wound environment. Notably, treatment with the rhCol III-EVs hydrogel led to a marked reduction in the inflammatory cytokine IL-6, along with elevated levels of the proliferation marker Ki67 and angiogenic indicators CD31 and α-SMA, indicating its role in accelerating the wound-healing process [50]. In a separate study, Munyemana et al. created porous, spherical hybrid nanomaterials using recombinant collagen combined with calcium carbonate. The recombinant collagen played a key role in shaping the nanoparticles and influencing their crystallinity, likely because its charged amino acids interact with calcium ions and help control the gradual formation process. These hybrid materials demonstrated excellent drug-loading capacity and released drugs in response to pH changes, making them promising candidates for drug delivery systems. They also showed strong biocompatibility in laboratory tests. Additionally, recombinant collagen can be produced in large quantities with high purity and consistency, making it a dependable and effective scaffold for designing innovative organic–inorganic nanomaterials for biomedical and therapeutic use [51]. Kong and colleagues designed a hydrogel made from recombinant type III collagen (rHCIII) to serve as a delivery platform for human adipose-derived stem cells (hADSCs), aiming to improve healing in diabetic wounds. This hydrogel created a supportive environment at the wound site, helping the stem cells stay alive and active for up to three weeks in lab tests. In animal studies, the system enhanced stem cell retention, amplified their therapeutic benefits, and significantly accelerated tissue regeneration and wound healing in diabetic mice [52]. In regenerative medicine, one strategy for repairing tissue defects involves implanting medical devices that create a favorable microenvironment for host cells, encouraging ECM secretion and tissue formation. In skin tissue engineering, both natural materials like collagen and HA and synthetic polymers such as PLA and PGA have been explored. Among them, recombinant humanized collagen offers customizable amino acid sequences and beneficial biological functions while minimizing risks like viral transmission and immune response. In this study, a skin damage model was used to evaluate recombinant type III collagen (rhCol III), which was found to improve photoaged skin by increasing collagen content, enhancing elasticity, and reducing skin thickening, though only in a limited preclinical setting. However, these findings highlight the therapeutic potential of engineered proteins like rhCol III as next-generation biomaterials for cosmetic, non-invasive anti-aging and skin rejuvenation applications [53]. Table 1 recaps the applications described.

### 3.2. Elastin-like Polypeptides

Elastin is a vital extracellular matrix protein that imparts elasticity to tissues such as blood vessels, lungs, and skin. Its structure consists of insoluble fiber networks formed through hydrophobic interactions, featuring repetitive peptide sequences like VPGG, VPGVG, and APGVGV. Elastin plays a key role in cellular processes such as adhesion, proliferation, and migration, making it a promising candidate for biomedical applications [54]. However, its natural form poses challenges: it is water-insoluble, immunogenic when derived from animal tissue, and difficult to manipulate. Chemically synthesized elastin mimics are often expensive and structurally inconsistent, prompting interest in recombinant production methods [55]. A significant breakthrough came when Dan Urry and colleagues discovered that natural elastin contains multiple VPGVG repeats [56]. This finding led to the development of elastin-like polypeptides (ELPs)—recombinant protein polymers produced via gene synthesis, based on elastin’s hydrophobic repeat sequences [57]. Elastin-like polypeptides (ELPs) exhibit reversible phase transitions that mimic the behavior of native elastin by undergoing a temperature-dependent solubility change, known as the inverse transition temperature (Tt). Below the Tt, ELPs remain soluble in aqueous environments, while above this threshold, they aggregate into coacervates, allowing for controlled assembly and disassembly. This tunable property enables precise control over drug release profiles, while their genetically defined composition ensures high biocompatibility and low immunogenicity, making them ideal for therapeutic applications [57]. In genetic engineering, also if Pichia pastoris has been used for ELP expression, Escherichia coli remains the preferred system due to its simplicity and high yield [58]. ELPs are typically designed as oligomers of repeating pentapeptides with the sequence (Val-Pro-Gly-X-Gly), or VPGXG, where X can be any amino acid except L-proline. These synthetic sequences resemble natural elastin and partially replicate its mechanical and biological properties [59]. One of the most remarkable features of ELPs is their reversible phase transition in aqueous solution. Below a specific transition temperature (Tt), ELPs are soluble; above it, they aggregate and phase separate. This thermoresponsive behavior is influenced by factors such as the number, arrangement, and composition of repeat units, overall hydrophobicity, and conjugation with other biomolecules [60]. Researchers have developed strategies to fine-tune elastin properties and to further broaden their functionality. Indeed, ELPs have been engineered into multiblock copolymers, typically by linking hydrophobic and hydrophilic segments (e.g., [VPGIG]_n1_–[VPGSG]_n2_), or by adding a hydrophobic terminal block to promote self-assembly or enhance material performance [61]. Furthermore, ELPs have been functionalized with bioactive peptides to enhance their biological activity or engineered to create fusion proteins by combining therapeutic proteins with ELPs. Elastin-based therapeutic systems enable efficient drug delivery due to their low toxicity, ability to self-assemble into nanoparticles, and targeted action [62]. For instance, attaching ELP to a tumor necrosis factor (TNF) antibody increases its molecular weight, which helps prevent renal filtration and extends its half-life from 28 min to around 11.4 h. Additionally, thanks to their temperature-sensitive properties, ELPs can form depot-like gel structures in the body, allowing for controlled, sustained release and prolonged therapeutic effects [63]. A similar approach was applied in a study involving interferon-alpha (IFN-α), a cytokine with well-established anti-tumor activity but limited clinical application due to its short circulation half-life. To address this, Gao et al. genetically fused ELP to the C-terminus of IFN-α, resulting in the IFN-α-ELP construct. This fusion retained the cytokine’s biological function in vitro, extended its half-life by approximately 30-fold, and significantly enhanced tumor accumulation compared to the native IFN-α [64]. In a follow-up study, the same research group evaluated the therapeutic potential of IFN-α-ELP in post-surgical immune-chemotherapy models of glioblastoma (GBM), a highly aggressive brain tumor. The fusion protein enabled sustained release through an ELP-based depot mechanism, exhibited zero-order release kinetics, and demonstrated improved pharmacokinetics and tissue distribution. It effectively penetrated brain tissues, triggered localized anti-tumor immune responses, and significantly reduced GBM recurrence [65]. To target acute myelogenous leukemia (AML), Alachkar et al. developed ELP-based fusion proteins directed at the FLT3 receptor, a key contributor to AML progression. These constructs showed sustained therapeutic efficacy in both in vitro and in vivo models. Additionally, the researchers created ELP-fused antibodies against CD99, another surface marker highly expressed in AML cells, which exhibited encouraging antileukemic effects [66]. Another notable development by Chilkoti et al. involved fusing a hexavalent DR5 death receptor agonist (DRA) with an ELP. This construct formed an in vivo depot for slow, controlled drug release. Remarkably, dosing could be reduced to once per week while maintaining anti-tumor efficacy and improving bioavailability [67]. In another approach, non-covalent drug binding was employed. MacKay et al. developed ELP-based gel nanoparticles that exhibit high-affinity non-covalent drug binding and integrin-mediated cellular uptake. This was the first study to demonstrate that ELP-Rapa effectively inhibits the mTOR signaling pathway in an HR+ breast cancer mouse model. Additionally, the formulation helped reduce drug-induced hemolysis, hepatotoxicity, and nephrotoxicity [68]. Dragojevic et al. engineered a glioblastoma-targeting drug delivery system using ELP’s thermal responsiveness. ELPs were fused with a cell-penetrating peptide (CPP) to facilitate cellular uptake, and Dox was conjugated via a pH-sensitive linker, ensuring drug release specifically in the acidic tumor environment. The CPP was genetically encoded into the ELP sequence, eliminating the need for additional chemical steps, while the pH-sensitive linker enabled precise, controlled drug activation [69]. Champion et al. enhanced the stability of ELP vesicles by incorporating p-azidophenylalanine (pAzF) into the ELP backbone. Upon UV exposure, this non-canonical amino acid promoted crosslinking, improving vesicle integrity. Water-soluble Dox was loaded by mixing it with the amphiphilic ELP and applying thermal triggers to induce self-assembly. Compared to unmodified constructs, these modified vesicles had lower transition temperatures and smaller sizes. In HeLa cells, the ELP-Dox conjugates successfully delivered and released cytotoxic Dox. Additionally, modifying the ELP sequence to include more hydrophobic residues slowed drug release and allowed more efficient encapsulation—even for drugs with lower hydrophobicity [70]. Kelly et al. explored a different application of ELPs by developing a thermoresponsive drug depot for CpG oligodeoxynucleotides (CpG), used alongside radioactive iodine (^131^I) to treat metastatic breast cancer. CpG stimulates innate immunity by activating dendritic cells and enhancing antigen presentation. To increase CpG’s in vivo half-life, researchers created an ELP fused with 12 lysine residues (ELP-K12), which electrostatically bound CpG and formed a depot upon intratumoral injection. This enabled sustained CpG release for up to three weeks, significantly inhibiting tumor growth and spread. Additionally, ELP was modified with tyrosine residues to allow radiolabeling with ^131^I, forming a stable compound that retained radioactivity for over 60 days without leakage at the tumor site [71]. For a further therapeutic application, a novel ELP nanofiber incorporating the REDV (arginine–glutamic acid–aspartic acid–valine) sequence was designed to enhance vascular graft performance. Compared to unmodified ELPs, RGD-ELPs, collagen, and glass, REDV-ELP nanofibers showed reduced platelet adhesion, improved endothelial cell attachment and proliferation, and supported smooth muscle cells in a contractile state—making them ideal for small-diameter vascular grafts [72]. Moreover, recent studies have revealed elastin’s role in promoting elastin and collagen fiber formation at wound sites, helping reduce scarring. Building on this idea, Feng et al. engineered a strong elastin-silk-like recombinant protein using *E. coli*, combining it with a nanobacterial cellulose layer to produce a bilayer skin substitute with excellent mechanical and antibacterial properties [73]. Similarly, Chen and colleagues developed a recombinant human collagen–elastin scaffold demonstrating improved membrane durability and supporting effective skin regeneration [74]. These materials show their properties by promoting wound healing in vitro and in vivo. Table 2 summarizes the described applications.

### 3.3. Silk-like Polymers

Another category of genetically engineered materials is silk-like polymers (SLPs), which are composed of repeating silk-like segments with the sequence Gly–Ala–Gly–Ala–Gly–Ser. Silk itself is a fibrous protein produced by spiders and insects like silkworms. Naturally occurring silks come in various forms and are known for their outstanding mechanical properties, including high strength, flexibility, biocompatibility, and biodegradability [75,76]. For industrial bioproduction of genetic-engineered silk, *Escherichia coli* is one of the most widely used organisms due to economically viable cell growth, high cell density, ease of manipulation, and it is often also used for proof of concept at laboratory scale. *E. coli* has also been used for recombinant spider silk production [77]. Genetically engineered silk-like polymers have primarily focused on designs based on the repeated sequences [GGAGQGGYGGLGSQ-GAGRGGLGGQGGAG] and [GPGGYGGPGQQGPGGYAPGQQPSGPGS], which are derived from the silk produced by the major ampullate glands 1 and 2 of *Nephila clavipes*, respectively [78]. Various modifications of these base sequences have also been identified. Some of these changes were used to regulate the degree of crystallinity, while others were introduced to add functionality to the polymers, such as incorporating RGD sequences to promote cell attachment. These genetically engineered silk-like polymers are typically regarded as block copolymers, consisting of highly conserved repeating units of short amino acids with small side chains that form hydrophobic blocks alongside shorter sequence segments [78,79]. Since polymers made solely of silk blocks have very low solubility in water and limited flexibility, combining them with elastin-like blocks (SELPs—silk-elastin-like polymers) through copolymerization enhances their flexibility and water affinity, while also decreasing overall crystallinity [32]. These characteristics are particularly important for purifying protein polymers and developing pharmaceutical formulations [80,81,82]. For example, Florczak et al. studied the effectiveness of H2.1-functionalized MS1 silk gel in a live mouse model of Her2-positive breast cancer. The results were consistent with earlier lab studies on human Her2-positive cancer cells: The H2.1MS1 silk gel particles loaded with doxorubicin selectively killed Her2-positive mouse cancer cells and significantly reduced tumor size over 20 days, with stronger effects at higher doses. Moreover, this drug delivery system caused less damage to non-target cells compared to free doxorubicin, as the treated mice maintained their body weight. In a separate experiment, unloaded MS1 spheres showed no toxicity when given to healthy mice. Although the presence of the H2.1 targeting peptide influenced how the particles were distributed in the body—resulting in more particles found in organs than non-targeted MS1 spheres—detailed examination of these organs revealed no structural damage [83]. Besides relying on electrostatic interactions, a promising method to control drug binding and release is by directly attaching the drug covalently to the carrier. For example, Herold et al. chemically linked doxorubicin to the surface of silk particles made from eADF4(C16), a specially engineered negatively charged spidroin derived from the European garden spider, *Araneus diadematus*. The drug was attached using a pH-sensitive hydrazine linker. At a neutral pH of 7.4, the particles released very little drug over 48 h, but when the pH was lowered to 4, simulating acidic conditions, the particles released the entire drug load within 24 h. Lab tests using hydrazine-linked fluorescent dyes showed that drug release was triggered within 16 h after the particles were taken up by cells [84]. Similarly, in a study by Mulinti et al., enzyme-responsive nanospheres were developed using a recombinant spider silk copolymer with a thrombin-sensitive linker for targeted antibiotic delivery. These nanospheres released vancomycin in response to Staphylococcus aureus infection and showed effective antibacterial activity in lab and animal models, offering a promising approach to treat drug-resistant infections [85]. In addition, in a recent study by Lian et al., a novel drug delivery nanofibrous membrane was developed by electrospinning a blend of recombinant spider silk protein (rMaSp) and sodium hydrogen sulfide (NaHS). SrMaSp/NaHS nanofibrous membrane exhibits excellent hemocompatibility and cytocompatibility, while also enabling sustained release of H2S over an extended period. Additionally, when loaded with endothelial progenitor cells (EPCs) and applied in an in vivo skin wound model, the rMaSp/NaHS/EPC membrane significantly improved wound healing compared to membranes made of rMaSp alone or rMaSp/NaHS without cells [86]. Similarly, silkworm-derived silk fibroin was fabricated into 3D hydrogel scaffolds and coated with recombinant spidroins produced using an *E. coli* expression system, fused with a cell-binding motif from fibronectin, a growth factor (fibroblast growth factor), and an antimicrobial peptide. This bioactive silk composite enhanced cell adhesion, antimicrobial activity, and growth factor stimulation. It also supported the formation of a bilayered skin tissue construct in vitro, demonstrating its potential as an affordable material for wound dressings and skin substitutes [87]. The same research group published a study on recombinant spidroins incorporating a fibronectin motif. Using the same approach, they fabricated 3D hydrogel scaffolds with silkworm-derived silk fibroin as the base material and coated them with recombinant spidroin. Their findings indicated that spidroins with the fibronectin motif hold significant potential for the treatment of burn wounds [88]. In addition to developing new materials for tendon–bone interface applications, engineered recombinant spider silk proteins were modified with peptide tags derived from bone-specific noncollagenous proteins, known as SIBLING proteins—such as osteopontin and sialoprotein—which play key roles in collagen interaction and mineralization initiation. The resulting spider silk–SIBLING hybrid gels were evaluated, in vitro, for their mineralization capacity and cellular interactions. These materials exhibited enhanced calcium phosphate deposition when incubated with mineralization agents. In gradient films, MC3T3-E1 mouse preosteoblasts showed preferential adhesion toward regions enriched with the collagen-binding motif [89]. A notable recent advancement involved a scaffold made from recombinant spider silk derived from the consensus motif of the repetitive core region of the major ampullate silk fibroin 4 of the garden cross spider, A. diadematus. This recombinant protein, named eADF4 (C16), consists of sixteen repeats of the polypeptide module C (amino acid sequence: GSSAAAAAAAASGPGGYGPENQGPSGPGGYGPGGP). It features multiple carboxylic acid groups that can bind calcium ions, promoting mineralization. In vitro experiments with mesenchymal stem cells (MSCs) cultured on these hydrogel scaffolds demonstrated a significant increase in alkaline phosphatase (ALP) activity, highlighting its promise for bone tissue engineering applications [90]. Additionally, a scaffold was developed by cloning a chimeric protein that combines a spider silk-inspired domain (SGRGGLGGQGAGAAAAAGGAGQGGYGGLGSQGT), which acts as an organic framework to regulate material stability and enable versatile processing, with the hyaluronic acid (HA) binding domain VTKHLNQISQSY (VTK), which controls osteogenesis. These hydrogel scaffolds promoted the differentiation of bone marrow-derived human mesenchymal stem cells (hMSCs) into osteoblastic lineage cells [91]. Finally, an engineered construct was produced by combining decellularized extracellular matrix (dECM) derived from horse joint cartilage to support tissue regeneration, with a silk-elastin-like protein hydrogel that acts as a biological glue to ensure adequate adherence to the host tissue. The results demonstrated that both materials possess unique properties that can be exploited to create a tailored microenvironment conducive to cell growth and differentiation, providing proof of concept for the construct’s in vitro biological and mechanical efficacy. The SELP hydrogel exhibited notable physical behavior, including a high resistance to mechanical stress, which aligns with the physiological loads experienced during locomotion [92]. Table 3 recaps the described applications.

## 4. Polysaccharide-Based Hydrogels Obtained Through Microbial Fermentation

**Figure 3:**
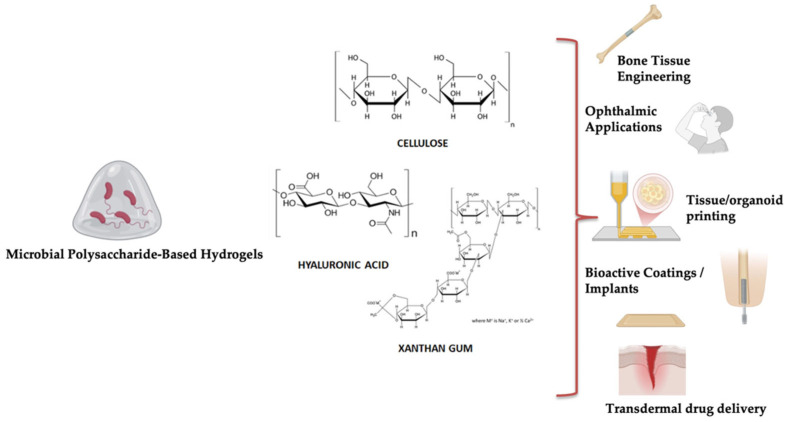
Biomedical applications of hydrogels derived from microbial polysaccharides.

In contrast to recombinant protein hydrogels, which rely on engineered structural proteins, the hydrogels presented in this section originate from microbially synthesized polysaccharides. These systems provide different structural architectures and functional properties that are particularly advantageous in soft tissue applications. Hydrogels derived from the controlled fermentation of sustainable biomaterials offer a promising alternative to conventional synthetic polymers, aligning with broader goals of environmental and biomedical sustainability. These bioengineered hydrogels not only provide mechanical support but also actively engage with the cellular microenvironment by delivering targeted biochemical signals and bioactive molecules. This facilitates precise regulation of key cellular processes such as proliferation, migration, and differentiation—processes essential for the reconstruction of complex tissues [93]. Central to the production of these next-generation hydrogels is the microbial biosynthesis of polysaccharides through the fermentation of carbohydrate-rich substrates. Selected microorganisms are employed to generate polymers with unique and tunable rheological, mechanical, and biological characteristics. Subsequent enzymatic engineering enables the selective functionalization of these polymers, introducing bioactive moieties that emulate the natural extracellular matrix and support the localized, controlled release of growth factors and other signaling molecules [94]. Natural polysaccharides obtained with this method, such as xanthan gum, cellulose, and hyaluronic acid—either used individually or in synergistic composite formulations (Figure 3)—are emerging as foundational components in the design of multifunctional hydrogel systems. Their complementary physicochemical properties contribute to hydrogels that are not only biocompatible and responsive to physiological conditions but also capable of orchestrating complex regenerative processes through spatially and temporally controlled bioactivity [95]. This innovative strategy is well-aligned with the One Health perspective. By utilizing renewable biological resources and eco-friendly production methods, the development of these hydrogels reduces ecological impact and minimizes dependence on petrochemical-based polymers. Moreover, their biodegradability and compatibility with living systems make them safer for clinical applications, while also reducing environmental burden.

### 4.1. Bacterial Cellulose

Bacterial cellulose (BC) is a macromolecule that shares a similar chemical composition with plant-derived cellulose, but it lacks compounds like lignin, pectin, and hemicellulose, which are typically found in plant cell walls [96]. However, BC differs structurally due to the unique arrangement of its fibers, resulting in enhanced mechanical strength, higher crystallinity, and freedom from associated plant polymers [97]. Structurally, BC is composed of β-1,4-linked glucan chains, stabilized by both intra- and intermolecular hydrogen bonds, and follows the general molecular formula (C_6_H_10_O_5_)_n_ [98]. The bacterium Acetobacter xylinum synthesizes two forms of cellulose: cellulose I, which appears as ribbon-like structures, and cellulose II, a more thermodynamically stable form. During biosynthesis, glucose chains are extruded from the bacterial cell wall as protofibrils, which then bundle into nanofibrils forming ribbon-shaped structures. These ribbons interweave into a dense, web-like network. The cellulose produced is rich in hydroxyl groups, which account for its strong water affinity, biodegradability, and its ability to undergo chemical modifications [98]. Because of its inherent characteristics—such as hydrophilicity, porosity, and biocompatibility—and its web-like network structure, bacterial cellulose is considered a promising material in the pharmaceutical field, particularly for enabling the controlled release of active compounds [98]. For example, bone tissue engineering strategies that utilize scaffolds are gaining increasing attention. This approach focuses on restoring bone by integrating biomaterials, living cells, and bioactive agents within a three-dimensional (3D) framework. The ultimate goal is to develop therapeutic constructs that not only support the repair and regeneration of injured bone tissue but also help prevent the spread of disease [99]. In this context, Cao and colleagues designed a nanocomposite hydrogel scaffold by incorporating oxidized BC with chitosan (CS) and nano-hydroxyapatite (nHA). Compared to scaffolds composed solely of CS and nHA, the new composite exhibited enhanced mechanical strength, a more favorable degradation profile, and greater water retention capability. When evaluated using MC3T3-E1 cells—a mouse-derived osteoblast precursor cell line—the scaffold demonstrated excellent biocompatibility and promoted higher levels of cell proliferation. Furthermore, in vivo experiments using a rat calvarial defect model confirmed the scaffold’s potential to support new bone formation [100]. In a separate study, Zhu et al. successfully developed a BC-based composite scaffold enhanced with CS and alginate (Alg). The resulting hydrogel scaffold formed a dense fibrous network and showed favorable swelling characteristics along with a suitable degradation rate. Additionally, this gel demonstrated excellent capabilities in promoting apatite formation, supporting cell compatibility, and facilitating protein adsorption and controlled release [101]. Promising developments have also emerged in cartilage tissue engineering using microbial cellulose. Li et al. developed a three-dimensional hierarchical porous scaffold composed of BC and decellularized cartilage extracellular matrix (DCECM) through a freeze-drying method, following chemical crosslinking with N-hydroxysuccinimide (NHS) and N-[3-(dimethylamino)propyl]-N′-ethyl carbodiimide hydrochloride (EDC). This hydrogel scaffold supported strong adhesion and proliferation of rabbit-derived chondrocytes. In vivo testing using a rabbit cartilage defect model revealed that the composite scaffold significantly enhanced cartilage regeneration compared to unmodified BC. Its high water retention and strong hydrophilicity provided excellent elasticity and shape-memory characteristics under wet conditions, closely resembling those of natural cartilage [102]. Han and colleagues successfully created a novel composite made of BC and poly (vinyl alcohol) (PVA) as a promising alternative for corneal stroma replacement. This new material demonstrated notable improvements in optical clarity, water retention, structural morphology, surface functional groups, and porosity compared to pure BC hydrogel, making it a strong candidate for corneal stroma engineering. Both in vitro biocompatibility tests and in vivo experiments in rabbits showed that the BC/PVA construct maintained the corneal stroma’s integrity, stability, and transparency better than BC alone after implantation [103]. In addition, a study by Oran et al. demonstrated that a BC composite scaffold incorporating quince seed mucilage—a glucuronoxylan polysaccharide hydrogel—exhibited improved swelling properties and promoted greater fibroblast adhesion and proliferation [104]. Microbial bacterial cellulose is also used in transforming drug delivery systems. In line with this, bacterial cellulose gel nanoparticles (BCNPs) were produced as a sustainable and eco-friendly platform for drug delivery. Characterization showed that prolonged culture time increased crystallinity and particle size. The BCNPs were thermally stable up to 90 °C and demonstrated successful loading and sustained release of a model drug, bovine serum albumin (BSA). These findings highlight BCNPs as a biodegradable, biocompatible, and green nanotherapeutic system with potential for scalable nanomedicine applications [105]. Zahel et al. investigate a new strategy for incorporating poorly water-soluble glucocorticoids into bacterial cellulose hydrogel. Five different microemulsion systems containing either hydrocortisone or dexamethasone were formulated and thoroughly characterized, confirming their uniform microstructure, biocompatibility, and storage stability. Transmission electron microscopy verified successful integration of these formulations into bacterial cellulose, with even water-in-oil (*w*/*o*) microemulsions showing uniform distribution. Drug permeation through Strat-M^®^ membranes was found to be both high and controllable, and in vitro assays confirmed that the permeated glucocorticoids maintained their anti-inflammatory effects [106]. Table 4 recaps the described applications.

### 4.2. Xanthan Gum

Xanthan gum (XG) is a high-molecular-weight heteropolysaccharide produced by the Gram-negative bacterium Xanthomonas campestris through aerobic fermentation of carbohydrate substrates. Valued for its unique rheological properties, biocompatibility, biodegradability, and ease of chemical modification, XG is a highly versatile biopolymer employed across numerous industries, ranging from food and cosmetics to pharmaceuticals. Recently, scientific interest has focused on its application in regenerative medicine. The intrinsic features of XG, such as environmental stimulus responsiveness and the ability to integrate with other biomaterials, make it an ideal candidate for designing advanced therapeutic systems, including injectable hydrogels, multiphasic scaffolds, bioinks, and controlled release platforms [107]. Specifically, XG-based hydrogels have demonstrated promising applications in bone and cartilage tissue regeneration due to their ability to mimic the natural extracellular matrix and support cellular proliferation and differentiation. Furthermore, XG can be chemically modified to enhance its mechanical and biological properties, rendering it suitable for specific tissue engineering applications [108]. Structurally, xanthan gum consists of a β-(1→4)-linked D-glucose backbone like cellulose. Every second glucose residue carries a lateral trisaccharide branch composed of an internal mannose linked α-(1→3) to the backbone, a glucuronic acid linked β-(1→2) to the mannose, and a terminal mannose linked β-(1→4) to the glucuronic acid. The internal mannose can be O-acetylated at the C6 position, while the terminal mannose is frequently pyruvylated between C4 and C6, introducing negatively charged groups that influence the polymer’s electrostatic and conformational behavior [109]. In aqueous solution, XG can adopt two main conformations: an ordered helical structure stabilized by hydrogen bonds and charge interactions, and a disordered, flexible single spiral form. The transition between these conformations depends on environmental parameters such as temperature, pH, and the presence of multivalent cations like Ca^2+^, Mg^2+^, and Fe^3+^. From a rheological perspective, XG exhibits pseudoplastic (shear-thinning) behavior, meaning its viscosity decreases with increasing shear rate. This is attributed to polymer chain alignment in the flow direction and temporary disruption of interchain interactions, facilitating material flow. Even at low concentrations, XG imparts high elasticity and viscosity due to electrostatic repulsion from pyruvate and acetyl groups, promoting polymer chain expansion [110]. The biotechnological production of xanthan gum (XG) is governed by a gene cluster known as the gum operon (from gumB to gumM), which encodes enzymes involved in the synthesis of sugar precursors, polymer assembly, and secretion [111]. Industrial-scale production of XG can be optimized both in terms of yield and polymer properties. The culture medium for Xanthomonas campestris includes carbon sources (e.g., glucose, sucrose, molasses), nitrogen sources (such as ammonium nitrate, peptone, yeast extract), mineral salts, and trace elements. The choice of carbon source significantly affects xanthan gum yield: sucrose tends to provide higher yields compared to glucose, which produces a slightly lower polymer amount. Additionally, controlled addition of certain organic acids—such as succinate, pyruvate, and α-ketoglutarate—can further stimulate production, although excessive concentrations may be inhibitory. Variations in culture medium composition not only influence polymer quantity but also modulate rheological and structural properties of the gum, potentially impacting its industrial and biomedical applications [112]. Furthermore, fermentation conditions such as temperature, pH, and aeration rate directly affect the viscoelastic properties of the produced XG [113,114]. The production and recovery process can significantly impact the environmental footprint of the polymer, motivating the adoption of alternative substrates and greener processes. Examples of sustainable substrates include agro-industrial residues such as whey and soybean biodiesel byproducts, which have proven effective for producing XG with rheological properties comparable to those from conventional sugars [115,116]. Additional biotechnological strategies to improve sustainability and economic efficiency include co-fermentation with engineered bacteria and the use of genetically modified X. campestris strains to increase production yield, modulate polymer functional properties, and avoid the use of alcoholic precipitants [117,118]. Thanks to its biocompatibility, stability, and ability to form highly viscous solutions, xanthan gum (XG) is widely used across numerous industrial sectors and represents a promising platform for the development of advanced biomedical devices. For example, a biodegradable, injectable polymeric liposomal hydrogel was developed based on liposomes containing xanthan gum (XG) modified with aldehyde groups and phosphatidylethanolamine. The chemical crosslinking of the hydrogel occurs via a Schiff base reaction between the aldehyde groups of the modified XG and the amine groups present on the liposomes, forming a stable and biocompatible network [119]. In parallel, 3D bioprinting approaches have utilized xanthan gum modified with methacrylate groups (XGMA) for the fabrication of hydrogel scaffolds specifically designed for post-traumatic cartilage therapy. In this case, functionalization improved the viscoelasticity of the bioink, its printability, and the mechanical properties of the resulting constructs. Additionally, these hydrogels demonstrated antioxidant capacity, protecting cells from oxidative stress, and exhibited high chondrogenic activity in vitro, making them suitable for advanced cartilage regeneration applications [120]. The integrated biotechnological approach has further promoted the development of innovative solutions combining functionalized polysaccharides and other components in synergistic systems aimed at enhancing cartilage regeneration. An example includes hybrid hydrogel scaffolds of XG and chitosan, used in conjunction with human amniotic fluid-derived mesenchymal stem cells stimulated with TGF-beta 3. These systems showed a high capacity to promote extracellular matrix formation and differentiation into chondrocytes in vitro, representing a promising strategy for treating cartilage lesions and for joint regenerative medicine [121]. XG has also been investigated in bone regenerative medicine. For instance, injectable hydrogels based on xanthan gum and gellan gum (XG/GG) containing chitosan nanoparticles and a dual growth factor system (bFGF/BMP7) were developed. This approach significantly enhanced the proliferation and differentiation of fetal osteoblastic cells, with increased calcium deposition and alkaline phosphatase activity compared to hydrogels containing a single growth factor [122]. Dense composite gel membranes composed of XG, chitosan, and hydroxyapatite have been developed for guided bone regeneration. The addition of hydroxyapatite improves bioactivity and mechanical properties, promoting adhesion and proliferation of mesenchymal stem cells in vitro. These materials show high potential for regenerative applications, including periodontal treatments, due to the synergy among the viscoelastic properties of XG, the mucoadhesive and antimicrobial capabilities of chitosan, and the osteoconductive potential of hydroxyapatite [123]. Formulations based on the combination of polysaccharides have shown great promise in vascular engineering. In particular, composite hydrogels of xanthan gum (XG) and alginate improve both the mechanical properties and biocompatibility of vascular grafts, such as expanded polytetrafluoroethylene (ePTFE). While ePTFE is widely used, it faces issues at small diameters due to thrombosis and poor endothelialization caused by suboptimal mechanical properties. Integration with XG-alginate hydrogel leverages alginate’s ability to form stable gels and XG’s promotion of endothelialization and mechanical reinforcement in vitro, making this combination suitable for vascular regenerative medicine applications, although in vivo validation is pending [124]. A similar approach using XG has been explored in dermatology, where this biopolymer, combined with natural polysaccharides such as konjac glucomannan (KGM) or chitosan, has shown promising results in skin wound healing. Bioactive gel membranes based on XG and chitosan support mesenchymal stromal cell proliferation and promote dermo-epidermal regeneration. These membranes also possess physical and biological properties favorable for tissue repair, suitable as dressings for chronic wounds and burns [125]. Likewise, thermo-reversible hydrogels formulated with XG and KGM exhibit physicochemical and biological features compatible with tissue regeneration, including high hydrophilicity, excess exudate absorption, and maintenance of a moist environment—critical conditions for effective wound healing. These hydrogels support cell adhesion, migration, and proliferation, suggesting potential use as smart dressings for acute and chronic skin lesions [126]. Due to its excellent physicochemical properties and biocompatibility, XG is also emerging as a versatile material for regenerating complex tissues such as the nervous system and cardiac tissue. Recent studies highlight XG’s potential in advanced systems for nerve injury repair, where structural and functional complexity requires materials that provide multiple guidance cues and a favorable microenvironment for cell regeneration. For example, a gellan-xanthan hydrogel conduit enriched with electrospun nanofibers loaded with regenerative factors like NGF, N-acetylcysteine, and magnesium oleate has shown efficacy in repairing sciatic nerve lesions with 10 mm gaps, improving functional recovery and reducing muscle atrophy compared to empty conduits. This approach holds promise for future studies on more extensive peripheral nerve injuries and longer recovery times [127]. Simultaneously, a composite bioink based on XG and methacrylated gelatin (GelMA) was developed to support differentiation of human induced pluripotent stem cells (hiPSC) into functional cardiomyocytes. This gel-like bioink, optimized for viscosity and 3D printability, has an elastic modulus comparable to cardiac tissue, fostering an ideal environment for cardiac cell growth, maturation, and spontaneous contraction [128]. A recap of the described applications is available in Table 5.

### 4.3. Hyaluronic Acid

Hyaluronic acid (HA) is a high-molecular-weight glycosaminoglycan found in the connective tissues of vertebrates, although it can also be biosynthesized by certain microorganisms [129]. The chemical structure of HA consists of repeating disaccharide units of D-glucuronic acid and N-acetyl-D-glucosamine, linked via alternating β(1→3) and β(1→4) glycosidic bonds. Unlike other glycosaminoglycans, HA is characterized by a linear, non-sulfated structure, which imparts unique physicochemical properties such as high water retention capacity, viscoelasticity, and biocompatibility—features that make it particularly suitable for biomedical and cosmetic applications [130]. The molecule exhibits a relatively rigid structure due to the stable configuration of its monomeric units and the formation of intra- and intermolecular hydrogen bonds. At physiological pH, the carboxylic groups of HA are ionized, imparting a negative charge to the molecule that facilitates interactions with cations—crucial for maintaining structural stability within tissues. This feature also enables HA to bind large amounts of water molecules through hydrogen bonding, resulting in molecular volume expansion and the formation of a hydrated network that contributes to the viscoelastic properties of the extracellular matrix (ECM) [131]. From a rheological perspective, HA solutions exhibit pseudoplastic behavior, characterized by a decrease in viscosity with increasing shear rate (shear-thinning). These characteristics play a critical role in HA’s functional performance in tissues and biomedical applications, where the modulation of viscosity and cohesiveness is essential for the polysaccharide’s effectiveness as an ECM component and biomaterial [132]. The molecular weight of HA is a key determinant of its biological functions, which can be broadly categorized into two main roles: as a structural component of the extracellular matrix (ECM) and as a cell signaling mediator. High-molecular-weight HA exhibits structural, lubricating, and space-filling properties due to the ability to form a highly hydrated, dynamic network that organizes proteoglycans and other matrix components. This contributes to tissue shape, turgidity, and integrity, while also dampening mechanical stress. Furthermore, these large polymers exhibit anti-angiogenic, anti-inflammatory, and immunosuppressive properties. In contrast, low-molecular-weight HA fragments penetrate tissues more effectively and function as pro-inflammatory, immunostimulatory, and pro-angiogenic signals. They promote neovascularization and tissue repair. Intermediate molecular weight fractions are involved in specific physiological processes such as wound healing, ovulation, and embryogenesis, and demonstrate good transdermal penetration capacity [133]. HA participates in a wide range of physiopathological processes, including embryonic development, tissue homeostasis, cell motility, inflammation, tumor progression and metastasis, hypoxic response, angiogenesis, and especially cutaneous wound healing. This versatility is enabled by HA’s function as a bioactive signaling molecule, interacting with specific cell surface receptors, the most prominent of which are RHAMM (CD168) and CD44 [134]. Initially, hyaluronic acid (HA) was exclusively extracted from animal tissues, such as rooster combs and fisheyes. However, this method presented several disadvantages, including harsh extraction conditions, high purification costs, risk of viral contamination, and potential immunogenicity due to residual animal-derived components. Furthermore, the quality of the final product varied depending on the age and species of the animal source, and significant product loss occurred due to the action of endogenous hyaluronidases [135]. To overcome these limitations, industrial HA production has progressively shifted toward microbial fermentation, which is now the predominant manufacturing method. This is made possible by wild-type bacterial strains, such as *Streptococcus equisimilis*, *S. pyogenes*, *S. uberis*, and particularly *Streptococcus equi* subsp. *Zooepidemicus* [136,137] or recombinant microbial platforms including *Lactococcus lactis*, *Enterococcus faecalis*, *Escherichia coli*, *Bacillus subtilis*, *Streptomyces albulus*, *Corynebacterium glutamicum*, *Agrobacterium* sp., *Saccharomyces cerevisiae*, and *Pichia pastoris* [136,137]. This biotechnology enables a more efficient, safer, and standardized HA production process, significantly reducing the risk of viral contamination and minimizing environmental impact [138]. To further expand the versatility of hyaluronic acid (HA) and tailor it to specific biomedical applications, a wide range of strategies has been developed to enhance its stability, functionality, and ability to deliver active molecules in a targeted and efficient manner through chemical modifications or interactions with other polysaccharides. For instance, blending HA with natural gums results in composites with improved viscosity and adhesiveness, which are advantageous in both cosmetic and pharmaceutical applications. Furthermore, HA can form polyelectrolyte complexes with positively charged polysaccharides such as chitosan, yielding controlled drug delivery systems in the form of nanoparticles or hydrogels [139]. In recent years, HA has gained growing interest in regenerative medicine, owing to its ability to modulate cell signaling, migration, proliferation, and differentiation. Its biodegradability also makes it a valuable temporary ECM substitute for tissue engineering. For example, HA-based hydrogel scaffolds have been developed for the support and implantation of engineered tissues in therapeutic applications such as bone, nerve, brain, and muscle regeneration, as well as for cell delivery [140]. Similarly, HA complex gel structures were fabricated through additive manufacturing technologies like electrohydrodynamic (EHD) processes and 3D bioprinting [141]. In particular, electrospinning enables the production of hydrogel fibers with controlled morphology [142], and needle-free electrospinning has yielded HA/chitosan gel membranes with excellent stability and biocompatibility [143]. Electrospraying, on the other hand, is used to produce microparticles or microgels suitable for controlled drug release and for generating cell-laden microgels capable of replicating specific tissue microenvironments [144]. In parallel, 3D bioprinting has emerged as an advanced tool for fabricating customized scaffolds, using HA gel-based bioinks combined with other biopolymers [145]. This approach has been applied to produce HA-based medical devices—such as gels and wound dressings—that maintain a moist wound environment conducive to re-epithelialization and angiogenesis, with proven benefits for acute wound healing (e.g., burns) and chronic lesions (e.g., diabetic ulcers) [146]. In cartilage regeneration, HA is widely used in biomimetic hydrogels that support chondrocyte viability and stimulate ECM synthesis. Among the most promising are thermoresponsive hydrogels modified with N-isopropylacrylamide (NIPAM), which can be injected in liquid form and gel in situ at body temperature, enabling minimally invasive procedures and showing significant efficacy in osteoarthritis treatment [147]. As a natural component of synovial fluid, HA plays a key role in joint homeostasis, contributing to lubrication and shock absorption. Consequently, it is successfully used in viscosupplementation therapies, involving intra-articular injections to restore the viscoelastic properties of synovial fluid, reduce inflammation, limit cartilage degradation, and stimulate cartilage matrix synthesis. These treatments are particularly effective in knee osteoarthritis, showing pain relief, improved joint mobility, and prolonged reduction in stiffness [148]. Similarly, HA has demonstrated strong potential in bone regeneration, particularly in combination with calcium phosphates, bioactive nanoparticles (e.g., hydroxyapatite), or osteogenic growth factors (e.g., BMPs), to create composite hydrogels that promote osteogenesis and vascularization [149]. These systems leverage both the mechanical and signaling properties of HA and the inorganic components to guide cell proliferation and differentiation at the injury site. Additional innovative strategies are under investigation to support the regeneration of complex tissues. For example, HA is being explored for myocardial regeneration, where injectable HA-based hydrogels can encapsulate mesenchymal stem cells (MSCs) and promote their adhesion and in situ differentiation [150]. Promising results have also been achieved in nerve tissue repair, where HA-based scaffolds stimulate axon growth and functional recovery in animal models of spinal cord injury [151]. Finally, with the advent of biofabrication, HA has become a key material for the creation of tissues and organoids. HA-based bioinks, with tunable rheological properties and photocrosslinking capabilities, enable the 3D printing of complex, viable, and functional cellular constructs, opening new frontiers in personalized medicine [151]. The described applications are summarized in Table 6.

## 5. Comparison Between Engineered Protein-Based and Microbial Polysaccharide-Based Hydrogels

Although both recombinant protein-based and microbial polysaccharide-based hydrogels are classified as bioengineered, sustainable materials, they differ markedly in origin, structure, and functional properties. Protein-based hydrogels—such as those derived from recombinant collagen, elastin, or silk—are typically designed to mimic the extracellular matrix (ECM), providing great mechanical strength, precise tunability, and bioactivity at the molecular level. These systems are suited for tissue engineering and regenerative medicine applications where mechanical performance and biological signaling are critical [10]. On the other hand, microbial polysaccharide-derived hydrogels, such as bacterial cellulose, xanthan gum, and hyaluronic acid, are valued for their natural gelling behavior, excellent water retention, biocompatibility, and ease of mass manufacturing from renewable substrates. These materials are particularly well-suited for topical treatments, wound dressings, and soft tissue scaffolding, where strong hydration and swelling properties are desirable. Highlighting this contrast is key to understanding how the biosynthetic origin shapes not only the physical and biological characteristics of the hydrogel but also its preferred biomedical use case. Table 7 acts as a comparative overview of recombinant and microbial-derived hydrogels for biomedical applications.

## 6. Future Perspectives and Conclusions

The utilization of sustainable hydrogels for medical applications is set to change significantly, thanks to ongoing advances in biomaterials science and a rising realization of the interconnection of human, animal, and environmental health under the One Health concept. Looking ahead, numerous significant themes are predicted to affect the future of this multidisciplinary area. First, the development of smart, stimuli-responsive hydrogels capable of dynamically responding to their biological surroundings is a potential area. Such systems, which respond to stimuli such as pH, temperature, or enzyme activity, can enable precise, on-demand medicinal delivery, increasing efficacy while decreasing side effects [152]. Simultaneously, the incorporation of sustainable concepts in hydrogel synthesis is crucial. Employing green chemistry approaches, which include renewable biopolymers, environmentally friendly solvents, and energy-efficient procedures, not only decreases environmental impact but also assures that the materials are biocompatible and safe for medicinal applications [153]. This is especially relevant when contemplating the translation of hydrogel technologies across the various biological systems covered by One Health. Furthermore, improvements in biofabrication and 3D bioprinting techniques bring great opportunities for developing sophisticated, patient-specific hydrogel-based constructions for regenerative medicine [154]. These technologies provide fine spatial control over scaffold design and biochemical signals, making tissue engineering applications advantageous for both human and veterinary medicine. Addressing the challenges of regulatory approval and scalable manufacturing is another vital aspect for future progress. Standardized characterization protocols, long-term biocompatibility data, and robust quality control measures will be required for clinical translation and general acceptance. Moreover, expanding efforts that explicitly involve multispecies and ecosystem-level perspectives will be extremely important. Future hydrogel technologies that embrace the holistic vision of One Health can help to combat zoonotic diseases, environmental contamination, and other health challenges at the human–animal–environment interface. However, a comprehensive assessment of the environmental sustainability of hydrogels should also consider life cycle analysis (LCA). LCA is a standardized methodology for quantifying the cumulative environmental impacts associated with all stages of a product’s life—from raw material extraction to production, use, and disposal. Recent studies have applied LCA frameworks to biodegradable and bio-based polymers, offering insights into their carbon footprint, water consumption, and end-of-life fate [154]. Integrating LCA approaches in the development and selection of hydrogel materials will be essential to ensure their long-term sustainability across medical and environmental applications. When selecting an appropriate hydrogel platform for biomedical applications, several factors must be considered, including biocompatibility, mechanical behavior, degradation kinetics, and the specific biological or anatomical context. Recombinant protein-based hydrogels such as collagen, elastin, and silk provide precise control over structure and bioactivity, and are particularly suitable for long-term scaffolds in tissue regeneration. In contrast, microbial polysaccharides such as bacterial cellulose or xanthan gum provide scalable and biocompatible alternatives, although their mechanical performance and degradation can be limiting without further functionalization. Hyaluronic acid, which is already utilized clinically, is extremely adaptable for soft tissue and injectable applications but often requires chemical modification to manage its degradation rate and biofunctionality. In the end, the choice of hydrogel should align with the target tissue’s physical and biological requirements—e.g., high elasticity for vascular applications, slower degradation for bone regeneration, or fast-resorbing systems for drug delivery. The integration of engineered hydrogels with application-specific design principles remains a key challenge for future translational success. In conclusion, sustainable hydrogels represent a significant advancement in the field of biomedical materials, offering a promising alternative to conventional synthetic systems. Their development through genetic engineering and microbial fermentation allows for the production of biocompatible, biodegradable, and structurally versatile platforms that meet contemporary demands for both therapeutic performance and environmental responsibility. In medical contexts, sustainable hydrogels have demonstrated wide-ranging utility, extending beyond traditional drug carriers to serve as active scaffolds in regenerative medicine, wound repair, and tissue engineering. Their high water content, structural mimicry of the extracellular matrix, and ability to modulate biomechanical and biochemical signals make them ideal platforms for supporting cell adhesion, proliferation, and differentiation. These properties are particularly advantageous in the treatment of chronic wounds, musculoskeletal injuries, and degenerative diseases, where conventional therapies often fall short. Furthermore, the capacity to fine-tune hydrogel architecture—through crosslinking density, degradation rate, and responsiveness to physiological stimuli—enhances therapeutic precision. This adaptability enables the design of systems tailored to specific anatomical sites or pathological conditions, expanding the scope of minimally invasive interventions. Equally significant is the sustainable foundation of these materials. Unlike petroleum-based polymers, recombinant and microbial-derived hydrogels are produced using renewable feedstocks, with lower energy inputs and minimal toxic byproducts. The incorporation of biodegradable components further ensures that these systems degrade into harmless substances post-application, reducing long-term environmental burden. These attributes are increasingly critical in the context of pharmaceutical pollution, where residues from drugs and delivery vehicles contribute to ecological degradation and antimicrobial resistance. The convergence of environmental support and materials innovation embodied by sustainable hydrogels exemplifies all the principles of the One Health paradigm. This integrative vision is increasingly reflected in international health policies and scientific agendas. Within this context, hydrogel technologies do more than address medical needs—they also contribute to systemic health goals, reinforcing the sustainability of healthcare systems and the ecosystems in which they operate. Looking forward, the continued refinement of hydrogel fabrication techniques, including advances in recombinant protein expression, enzymatic crosslinking, and 3D bioprinting, will open new frontiers in personalized and regenerative medicine. Collaborations across biotechnology, materials science, and clinical disciplines will be essential to fully harness the potential of these systems.

## Figures and Tables

**Table 1:** Applications of genetic-engineered collagen hydrogels.

Device	Applications	Key Features	Reference
Collagen (RCPhC1) modified with MA, NB, and SH	3D-printed tissue scaffolds	Suitable for two-photon polymerization (2PP); supports cell adhesion and proliferation in vitro; sub-micrometer resolution	[49]
Collagen (rhCol III) + MSC-EVs hydrogel	Wound healing and inflammation control	Modulates macrophage polarization (M2); reduces IL-6; increases Ki67, CD31, and α-SMA; supports angiogenesis and tissue repair (in vitro + in vivo)	[50]
Recombinant collagen + CaCO_3_ nanoparticles	Drug delivery systems	pH-responsive drug release; porous spherical nanostructure; high drug-loading; good biocompatibility; tunable crystallinity	[51]
Collagen (rhCol III) hydrogel + hADSCs	Diabetic wound healing	Prolongs stem cell survival; enhances retention and regeneration; improves healing in diabetic mouse models	[52]
Recombinant humanized type III collagen	Skin regeneration (photoaged skin)	Increases collagen content; improves elasticity; reduces dermal thickening; safe and customizable for regenerative applications	[53]

**Table 2:** Applications of genetic-engineered elastin hydrogels.

Device	Applications	Key Features	Reference
Elastin-like peptide (ELP)-TNF antibody	Anti-inflammatory/anti-tumor	Increased molecular weight; prolonged half-life (from 28 min to 11.4 h); depot formation	[63]
IFN-α-ELP fusion	Anti-tumor therapy	30-fold extended half-life; preserved bioactivity; enhanced tumor accumulation	[64]
IFN-α-ELP	Glioblastoma immune-chemotherapy post-surgery	Zero-order release; improved pharmacokinetics; brain penetration; anti-tumor immune response	[65]
FLT3-ELP fusion	Targeted therapy for AML	Sustained activity; FLT3 receptor targeting; efficacy in vitro and in vivo	[66]
CD99-ELP antibody fusion	AML treatment	Targeted CD99 on AML cells; notable antileukemic effects	[55]
ELP-DRA (hexavalent DR5 agonist)	Cancer therapy	Depot-forming; once-weekly dosing; improved bioavailability	[67]
ELP-Rapa nanoparticles	HR+ breast cancer	Non-covalent drug binding; integrin-mediated uptake; reduced systemic toxicity	[68]
ELP-CPP-Dox conjugate	Glioblastoma drug delivery	Thermal responsiveness; CPP-facilitated uptake; pH-sensitive linker for targeted drug release	[58]
UV-crosslinked ELP vesicles	HeLa cell cytotoxicity testing	pAzF for UV-induced crosslinking; thermal-triggered self-assembly; lower transition temp; improved delivery	[70]
ELP-K12 + CpG + ^131^I	Metastatic breast cancer immunoradiotherapy	CpG depot formation; 3-week sustained release; radiolabeled with ^131^I; long retention at tumor site	[71]
REDV-ELP nanofibers	Small-diameter vascular grafts	Reduced platelet adhesion; enhanced endothelial/smooth muscle cell interactions	[72]
Elastin-silk recombinant + cellulose	Skin regeneration/wound healing	Bilayer skin substitute; mechanical strength; antibacterial properties	[73]
Collagen-elastin recombinant scaffold	Skin regeneration	Durable membrane; supports regeneration and healing	[74]

**Table 3:** Applications of genetic-engineered silk hydrogels.

Device	Applications	Key Features	Reference
H2.1-MS1 silk gel with DOX	Her2-positive breast cancer treatment	Targeted delivery; reduced tumor size; low toxicity; preserved body weight in mice	[83]
Silk eADF4(C16)-DOX via hydrazine linker	pH-sensitive drug release	Stable at pH 7.4; complete release at pH 4; intracellular activation within 16 h	[84]
Spider silk copolymer with thrombin-sensitive linker	Targeted antibiotic delivery for *S. aureus* infections	Enzyme-responsive release; effective antibacterial activity in vitro and in vivo	[85]
Silk (rMaSp)/NaHS nanofibrous membrane	Wound healing	Sustained H_2_S release; good hemocompatibility; enhanced healing with EPCs	[86]
Silk fibroin hydrogel + recombinant spidroins with fibronectin, FGF, AMP	Skin substitutes and wound dressings	Enhanced cell adhesion; antimicrobial; bilayered tissue formation	[76]
Spidroins with fibronectin motif on silk fibroin hydrogel	Burn wound treatment	Enhanced regeneration; fibronectin-mediated cellular interaction	[87]
Spider silk–SIBLING hybrid gel	Tendon–bone interface engineering	Improved mineralization; specific cell adhesion; bone protein motifs	[89]
Silk eADF4(C16) hydrogel scaffold	Bone tissue engineering	Calcium-binding; increased ALP activity; MSC osteogenic differentiation	[90]
Spider silk–VTK chimera hydrogel	Osteogenesis and stem cell therapy	HA-binding domain; hMSC differentiation into osteoblasts	[91]
Silk dECM + SELP hydrogel	Cartilage tissue regeneration	Mechanical resilience; bioadhesion; supports cell differentiation	[92]

**Table 4:** Applications of cellulose hydrogels obtained by controlled fermentation.

Device	Applications	Key Features	Reference
BC/CS/nHA hydrogel scaffold	Bone tissue engineering	Enhanced mechanical strength, improved degradation profile, superior water retention, high cell proliferation, confirmed bone formation in vivo	[100]
BC/CS/Alg hydrogel scaffold	Bone tissue engineering	Dense fibrous network, good swelling and degradation behavior, excellent apatite formation, protein adsorption, and controlled release	[101]
BC/DCECM scaffold (NHS/EDC crosslinked)	Cartilage regeneration	Strong chondrocyte adhesion/proliferation, enhanced cartilage regeneration in vivo, high elasticity, water retention, shape-memory, mimics natural cartilage	[102]
BC/PVA composite	Corneal stroma replacement	Improved optical clarity, water retention, morphology, porosity; in vivo biocompatibility and transparency maintenance	[103]
BC/quince seed mucilage scaffold	Wound healing, tissue repair	Enhanced swelling properties, improved fibroblast adhesion and proliferation	[104]
BCNPs (BC nanoparticles)	Drug delivery systems	Biodegradable, thermally stable (up to 90 °C), increased crystallinity with time, sustained BSA release	[105]
BC with glucocorticoid microemulsions	Transdermal drug delivery	Uniform microstructure, high permeation through Strat-M^®^, preserved anti-inflammatory activity, stable formulations	[106]

**Table 5:** Applications of xanthan gum hydrogels obtained by controlled fermentation.

Device	Applications	Key Features	Reference
XG-based liposomal hydrogel	Injectable drug delivery, regenerative therapy	Biodegradable, chemically crosslinked via Schiff base reaction, stable, biocompatible	[119]
XGMA hydrogel scaffold (3D printed)	Post-traumatic cartilage therapy	Enhanced printability, viscoelasticity, antioxidant activity, chondrogenic potential	[120]
XG/chitosan hybrid hydrogel + hAF-MSCs + TGF-β3	Cartilage regeneration	Promotes ECM formation and chondrogenic differentiation, high bioactivity	[121]
XG/GG injectable hydrogel + CS NPs + bFGF/BMP7	Bone regeneration	Enhanced osteoblast proliferation/differentiation, high ALP activity, calcium deposition	[122]
XG/CS/hydroxyapatite gel membrane	Guided bone regeneration, periodontal repair	Improved bioactivity, mechanical strength, mucoadhesive, osteoconductive, antimicrobial	[123]
XG/alginate hydrogel	Vascular graft enhancement	Improved biocompatibility, mechanical properties, endothelialization of ePTFE grafts	[124]
XG/CS membrane	Skin wound healing	Supports stromal cell proliferation, dermo-epidermal regeneration, suitable for chronic wounds/burns	[125]
XG/KGM thermo-reversible hydrogel	Wound healing (smart dressing)	High hydrophilicity, moisture retention, exudate absorption, supports cell migration/proliferation	[126]
Gellan-XG hydrogel conduit + electrospun nanofibers + NGF/NAC/MgO	Peripheral nerve regeneration	Supports sciatic nerve repair (10 mm gap), reduces atrophy, enhances functional recovery	[127]
XG/GelMA composite bioink	Cardiac tissue regeneration	Mimics cardiac modulus, supports hiPSC differentiation into cardiomyocytes, spontaneous contractions	[128]

**Table 6:** Applications of hyaluronic acid hydrogels obtained by controlled fermentation.

Device	Applications	Key Features	Reference
HA-based hydrogel scaffolds	Bone, nerve, brain, muscle regeneration; cell delivery	Biocompatible, supports engineered tissue implantation and cell viability	[140]
HA complex gel structures (EHD, 3D printing)	Tissue engineering	Fabricated via EHD/3D bioprinting, customizable architecture	[141]
HA-based hydrogel fibers (electrospinning)	Controlled drug delivery, tissue engineering	Controlled fiber morphology, injectable forms, scalable	[142]
HA/Chitosan membranes (needle-free electrospinning)	Biomedical coatings, wound healing	Excellent stability, enhanced biocompatibility	[143]
HA microparticles/microgels (electrospraying)	Drug delivery, tissue mimicry	Enables controlled release, cell encapsulation for niche recreation	[144]
HA-based bioinks (3D bioprinting)	Customized tissue scaffolds	Printable, biocompatible, combined with other biopolymers	[145]
HA-based gels and wound dressings	Wound healing (acute/chronic)	Maintains moist environment, promotes angiogenesis and re-epithelialization	[146]
Thermoresponsive HA-NIPAM hydrogels	Osteoarthritis treatment	Injectable, in situ gelling, supports cartilage ECM synthesis	[147]
HA injections (viscosupplementation)	Knee osteoarthritis	Restores synovial fluid properties, reduces inflammation and stiffness	[148]
HA/CaP or HA/BMP composite hydrogels	Bone regeneration	Osteoconductive, promotes vascularization and osteogenesis	[149]
Injectable HA hydrogels + MSCs	Myocardial regeneration	Supports MSC adhesion, survival, and differentiation	[150]
HA scaffolds	Nerve regeneration	Stimulates axonal regrowth and functional recovery in SCI models	[151]
HA-based bioinks (photocrosslinkable)	Tissue/organoid printing	Tunable rheology, viable 3D constructs, enables personalized medicine	[151]

**Table 7:** Comparative overview of recombinant and microbial-derived hydrogels for biomedical applications.

Hydrogel Type	Origin	Biocompatibility	Mechanical Properties	Degradation Rate	Key Applications	Limitations
Recombinant Collagen	Recombinant (*E. coli*, yeast)	Excellent (widely validated in vivo)	Moderate (can be tuned)	Slow to moderate	Skin regeneration, wound healing	Potential cost, thermal instability
Elastin-like Polypeptides (ELPs)	Recombinant (*E. coli*)	Excellent	Highly elastic, tunable	Slow	Drug delivery, vascular tissue	Complex design, scale-up challenges
Silk-like Polymers (Spider Silk, SELPs)	Recombinant (*E. coli*, yeast)	Very good	High tensile strength	Slow	Bone, nerve, and skin regeneration	Limited biodegradability control
Bacterial Cellulose (BC)	Microbial (A. xylinum)	Excellent	High (wet state strength)	Very slow	Wound dressing, bone/cartilage repair	Poor degradability, no cell-adhesion motifs
Xanthan Gum (XG)	Microbial (X. campestris)	Good (in vitro and in vivo)	Soft, easily modifiable	Moderate to fast	Injectable scaffolds, nerve tissue	Low mechanical strength alone
Hyaluronic Acid (HA)	Microbial (recombinant or native)	Excellent (clinical use)	Soft, viscoelastic	Fast (hours–days)	Osteoarthritis, cartilage, ophthalmic	Rapid clearance, needs modification

## Data Availability

No new data were created or analyzed in this study.
